# Theoretical Analysis and Experimental Verification of the Stress and Strain of Axially Compressed Steel-Reinforced Concrete Columns under Long-Term Loads

**DOI:** 10.3390/ma15217630

**Published:** 2022-10-30

**Authors:** Weiwei Han, Cui Wang, Yigang Lv, Miao Su, Yuting Liu, Hui Peng

**Affiliations:** 1 National Engineering Research Center of Highway Maintenance Technology, Changsha University of Science & Technology, Changsha 410114, China; 2 School of Traffic & Transportation Engineering, Changsha University of Science & Technology, Changsha 410114, China; 3 School of Civil Engineering, Changsha University of Science & Technology, Changsha 410114, China; 4 Engineering Laboratory of Bridge Structure Safety Control of Hunan Province, Changsha University of Science & Technology, Changsha 410114, China

**Keywords:** steel reinforced concrete column, axial compression, stress and strain, calculation program, stress redistribution

## Abstract

The objective of this study is to provide a theoretical method to accurately calculate the stress and strain of steel-reinforced concrete (SRC) columns under long-term axial compression. First, considering the cross-sectional stress redistribution and the influence of each stress increment in the process, the theoretical formula of stress and strain under long-term loading was deduced. Then, the stress and strain calculation program of SRC columns under long-term axial compression was programmed by using object-oriented Visual C++ language. Finally, an experimental study on the long-term deformation performance of SRC axial compression columns was performed to validate the accuracy of the proposed theoretical method. By comparing the calculated results with the experimental results, the influence of steel bars on the long-term stress and strain of SRC columns under axial compression was analyzed and the corresponding long-term stress–strain variation law was studied. Results show that the changing trend of the long-term strain of plain concrete (PC) and SRC with loading time is basically the same, increasing rapidly in the first 270 days and gradually tending to be stable beyond 270 days. After 750 days, the maximum difference in the total strain between the PC columns and SRC columns reaches 26.60%, and the steel bars have a strong influence on the long-term strain of the concrete columns. The errors between the measured values of the two SRC columns, and the calculated results are 2.96% and 5.78%, respectively. Therefore, the derived stress–strain calculation formula and calculation program of SRC columns under long-term loads are accurate and reliable. When the loading time is 750 days, the tensile stress increment of 1.92 MPa and a compressive stress increment of 168.26 MPa are produced in concrete and steel bars. The long-term stress of concrete columns is markedly influenced by steel bars. In the first three years, the stress and strain of the concrete and steel bars develop rapidly and then gradually slow down.

## 1. Introduction

The shrinkage and creep of concrete are primary factors that affect the long-term deformation of concrete structures. However, many factors affect shrinkage and creep that result in the change law of shrinkage and creep being complex, time-varying, and random [1,2,3,4,5,6,7,8,9,10]. Many scholars have performed long-term theoretical and experimental research on concrete shrinkage and creep and proposed prediction models of concrete shrinkage and creep, considering different influencing factors [11,12,13,14]. To date, no theory has been developed that can completely and accurately explain or predict the shrinkage and creep characteristics of concrete.

Steel-reinforced concrete (SRC) columns are the most basic load-bearing members in various engineering structures, such as buildings and bridges. It is important to accurately analyze the vertical deformation of SRC columns under long-term loading and reduce the deformation and stress of the structure. Hamed Ehab et al. [15] established a mathematical model to consider the creep and geometric nonlinearity of SRC columns in the nonlinear range of stress, shrinkage, and aging. Numerical calculations indicated the importance of considering geometric nonlinearity and material nonlinearity in the creep analysis of concrete columns. Wu et al. [16] studied the difference in drying shrinkage deformation between partially enclosed steel-reinforced concrete (PSRC) columns and plain concrete (PC) columns due to different water diffusion by finite element analysis. In addition, they proposed an improved B3 model to predict the drying shrinkage of PSRC. Shariff Mohammad-Najeeb et al. [17] proposed an accurate method to estimate the time-varying strain in SRC members subjected to concentric axial compression using the creep flexibility and shrinkage strain information of corresponding plain concrete. The axial strain in concrete was assumed to be the sum of the shrinkage strain and creep strain. An Gyeong-Hee et al. [18] modified the existing FIB2010 and B3 models of SRC columns by inserting coefficients into the time function. The coefficients α and β were determined by the regression of the long-term deformation of SRC columns. Coefficient α is used to reflect the horizontal shift, and β is for the decrease of the slope; the authors concluded that the coefficients were primarily dependent on the geometry of the wide-flange steel. Chen et al. [19] performed a long-term axial load test study of short SRC columns, and their results showed that the age-adjusted effective modulus method can better simulate the deformation development of SRC columns under long-term axial loads using the shrinkage model of ACI 209R-92 and the creep model of CEB-FIP90. Zhang et al. [20] studied the effect of steel bars on the shrinkage strain of concrete by conducting 800-day shrinkage comparison tests on PC columns and SRC columns. By fitting the measured data, the influence coefficient of steel bars on the shrinkage strain of concrete was calculated, and the CEB-FIP model and the model of the China Academy of Building Research (CABR) were revised under the consideration of reinforcement. Zheng et al. [21] considered the stress redistribution of the section and used a program written in MATLAB to analyze the influence of the key parameters on the creep of axially compressed column concrete. They concluded that the ultimate creep value of the axial compression SRC column decreases with the prolongation of the loading age, the increase in the longitudinal compression steel reinforcement ratio, section size, and relative humidity, and the decrease in the concrete strength, initial stress level, and temperature. The relations between the stable ultimate creep value of the axial compression steel reinforced C20~C50 concrete column and each key parameter was fitted based on the simulation results. Zhou et al. [22] introduced a finite element step-by-step calculation method for creep effect analysis, in which the beam was regarded as a plain concrete structure without considering the restraint of its internal steel bars. However, the concrete structure is equipped with a certain amount of steel bars, which restrains the deformation of the concrete. Therefore, the existence of non-prestressed reinforcement has a strong influence on the long-term stress and long-term deformation of the section [23]. Based on the age-adjusted effective modulus method, Fu et al. [24] studied the initial elastic strain and shrinkage creep strain of SRC columns with different reinforcement ratios, as well as the elastic stress and shrinkage creep stress of each part of the material, but the influence of each stress increment in the process was not accurately considered. By analyzing the shrinkage and creep of SRC columns with different reinforcement ratios, the optimal reinforcement ratio of the columns was determined to be 2% to 3%.

In SRC columns subjected to long-term loads, if the bond performance between the steel bar and concrete is good, the long-term stress and strain of columns will be affected by the deformation coordination between the steel bars and concrete. The aging characteristics of shrinkage and creep of concrete will cause cross-section stress redistribution [25,26,27,28], and the constraint effect of steel bars on shrinkage and creep of concrete columns and stress redistribution between steel bars and concrete cannot be ignored [21,29,30,31,32,33,34]. Currently, most shrinkage prediction models and experiments do not consider the influence of steel bars, and the corresponding calculations are based on plain concrete [35,36]. When analyzing the shrinkage and creep effect of structures with neglecting the influence of steel bars on shrinkage and creep, errors are inevitably introduced.

To accurately calculate the stress and strain of SRC columns under long-term loading and the effect of cross-sectional stress redistribution and each stress increment on the long-term stress and strain, the theoretical calculation formula of stress and strain under long-term loading is derived in this paper. Using object-oriented Visual C++ language, the stress and strain calculation program of SRC columns under long-term axial compression is programmed to implement this calculation process. The influence of the steel bar on the stress and strain of concrete columns is analyzed, and the variation law of long-term stress and strain of SRC columns is studied to provide a reference for future research on the long-term deformation performance of SRC columns considering cross-section stress redistribution.

## 2. Theoretical Derivation

### 2.1. Basic Assumptions

**Figure 1:**
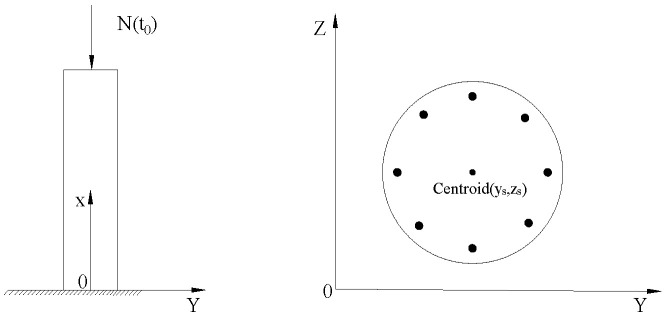
Schematic diagram of the axially compressed column.

At the time instant $t_{0}$, it is assumed that the area of axially compressed SRC columns is A; the centroid position of the cross-section is $\left(y_{s},y_{z}\right)$; the elastic moduli of concrete and ordinary steel bars are $E_{c}\left(t_{0}\right)$ and $E_{s}$, respectively; the reinforcement ratio is ρ; and the axial pressure at the centroid is $N\left(t_{0}\right)$. A schematic diagram of an axially compressed column is shown in Figure 1.

During all calculations, the following basic assumptions are used:

1. Concrete is a homogeneous and isotropic material, and has good working performance with steel bars without relative slip;
2. The plane section of the section is assumed to hold;
3. The steel bar is always elastic, regardless of creep and stress relaxation effects;
4. The assumption of the linear creep of concrete and the principle of superposition of strains are valid.

### 2.2. Calculation of Short-Term Stress and Strain

At the initial moment $t_{0}$, steel bars and concrete work together to bear axial pressure $N\left(t_{0}\right)$:

(1) $$N\left(t_{0}\right)=N_{c}\left(t_{0}\right)+N_{s}\left(t_{0}\right)$$

The short-term elastic strain $\varepsilon \left(t_{0}\right)$ at time $t_{0}$ is:

(2) $$\varepsilon \left(t_{0}\right)=\frac{N_{}\left(t_{0}\right)\;}{E_{c}\left(t_{0}\right)\cdot A_{c}+E_{s}\cdot A_{s}}=\frac{N_{}\left(t_{0}\right)\;}{E_{c}\left(t_{0}\right)\cdot A\left(1+\left(n-1\right)\cdot \rho \right)}\;$$

where $A_{c}$ and $A_{s}$ are the concrete area and steel bar area of the section, respectively; A is the total cross-sectional area; ρ is equal to $A_{s}/A$; and n is equal to $E_{s}/E_{c}\left(t_{0}\right)$.

The short-term elastic stress $\sigma_{c}\left(t_{0}\right)$ of concrete at time $t_{0}$ is:

(3) $$\sigma_{c}\left(t_{0}\right)=E_{c}\left(t_{0}\right)\cdot \varepsilon \left(t_{0}\right)=\frac{N\left(t_{0}\right)}{A\cdot \left[1+\left(n-1\right)\cdot \rho \right]}$$

Lastly, the elastic stress of steel bar $\sigma_{s}\left(t_{0}\right)$ at time $t_{0}$ is:

(4) $$\sigma_{s}\left(t_{0}\right)=E_{s}\cdot \varepsilon \left(t_{0}\right)=\frac{n\cdot N\left(t_{0}\right)}{A\cdot [1+(n-1)\cdot \rho ]}$$

### 2.3. Calculation of Long-Term Stress and Strain

Under long-term loading, SRC columns under axial compression will shrink and creep, which will lead to stress redistribution between longitudinal reinforced bars and concrete. Considering the redistribution of the cross-section stress and based on the linear superposition principle, the long-term stress–strain calculation formula is derived.

Under constant load, when the long-term loading time is $t_{i}\left(t_{i}>t_{0}\right)$, the cumulative strain increment of shrinkage and creep of the cross-section concrete is:

(5) $$\sum_{j=1}^{i}\Delta \varepsilon_{c}\left(t_{i},t_{j}\right)=\frac{\sigma_{c}\left(t_{0}\right)\cdot \left[1+\varphi \left(t_{i},t_{0}\right)\right]}{E_{c}\left(t_{0}\right)}+\frac{\sum_{j=1}^{i}\Delta \sigma_{c}\left(t_{i},t_{j}\right)\cdot \left[1+\chi \left(t_{i},t_{j}\right)\cdot \varphi \left(t_{i},t_{j}\right)\right]}{E_{c}\left(t_{0}\right)}-\varepsilon \left(t_{0}\right)+\varepsilon_{sh}\left(t_{i},t_{s}\right)-\varepsilon_{sh}\left(t_{0},t_{s}\right)\;=\frac{\sigma_{c}\left(t_{0}\right)\cdot \varphi \left(t_{i},t_{0}\right)}{E_{c}\left(t_{0}\right)}+\frac{\sum_{j=1}^{i}\Delta \sigma_{c}\left(t_{i},t_{j}\right)\cdot \left[1+\chi \left(t_{i},t_{j}\right)\cdot \varphi \left(t_{i},t_{j}\right)\right]}{E_{c}\left(t_{0}\right)}+\varepsilon_{sh}\left(t_{i},t_{s}\right)-\varepsilon_{sh}\left(t_{0},t_{s}\right)$$

where $\Delta \varepsilon_{c}\left(t_{i},t_{j}\right)$ is the marginal increment of strain caused by shrinkage and creep of concrete from $t_{j-1}$ to $t_{j}$; $\Delta \sigma_{c}\left(t_{i},t_{j}\right)$ is the marginal increment of stress caused by shrinkage and creep of concrete from $t_{j-1}$ to $t_{j}$; $\varphi \left(t_{i},t_{j}\right)$ is the creep coefficient with loading age $t_{j}$ and calculation age $t_{i}$; $\varepsilon_{sh}\left(t_{i},t_{s}\right)$ is the shrinkage strain at the calculated age $t_{i}$; $\varepsilon_{sh}\left(t_{0},t_{s}\right)$ is the shrinkage strain at age $t_{0}$; $\chi \left(t_{i},t_{j}\right)$ is the aging coefficient with loading age $t_{j}$ and calculation age $t_{i}$; $t_{s}$ is the concrete age (d) at the beginning of shrinkage; and $t_{0}$ is the loading age (d) of concrete.

If the aging coefficient is calculated accurately, the Dischinger method can be used. The dispatcher method establishes an incremental deformation coordination differential equation in time increments to solve the secondary internal force of structural creep. The expression is as follows:

(6) $$\chi \left(t_{i},t_{j}\right)=\frac{1}{1-e^{-\varphi \left(t_{i},t_{j}\right)}}-\frac{1}{\varphi \left(t_{i},t_{j}\right)}$$

According to the principle of mechanical balance, long-term loading to time $t_{i}$ should meet the following requirements:

(7) $$\Delta N_{c}\left(t_{i}\right)+\Delta N_{s}\left(t_{i}\right)=0$$

where $\Delta N_{c}\left(t_{i}\right)$ is the increment of the concrete internal force of the section in $t_{i}$ and $\Delta N_{s}\left(t_{i}\right)$ is the increment of the internal force of the steel bar in the section in $t_{i}$.

Equation (7) can be converted to:

(8) $$\sum_{j=1}^{i}\Delta \sigma_{c}\left(t_{i},t_{j}\right)A_{c}+\sum_{j=1}^{i}\Delta \sigma_{s}\left(t_{i},t_{j}\right)A_{s}=0$$

where $\sum_{j=1}^{i}\Delta \sigma_{c}\left(t_{i},t_{j}\right)$ is the cumulative increment of stress of concrete under long-term loading; $\sum_{j=1}^{i}\Delta \sigma_{s}\left(t_{i},t_{j}\right)$ is the cumulative increment of stress of steel bars under long-term loading; and $\Delta \sigma_{s}\left(t_{i},t_{j}\right)$ is the marginal stress increment caused by stress redistribution of steel bars from $t_{j-1}$ to $t_{j}$.

Due to the good working performances of concrete and steel bars, there is no relative slip when the long-term loading time is $t_{i}$:

(9) $$\sum_{j=1}^{i}\Delta \varepsilon_{c}\left(t_{i},t_{j}\right)=\sum_{j=1}^{i}\Delta \varepsilon_{s}\left(t_{i},t_{j}\right)$$

where $\sum_{j=1}^{i}\Delta \varepsilon_{s}\left(t_{i},t_{j}\right)$ is the cumulative increment of the strain of the steel bar when the long-term load is loaded to $t_{i}$, and $\Delta \varepsilon_{s}\left(t_{i},t_{j}\right)$ is the marginal increment of the strain caused by the stress redistribution of steel bars from $t_{j-1}$ to $t_{j}$.

Combining Equations (8) and (9) yields:

(10) $$\sum_{j=1}^{i}\Delta \sigma_{c}\left(t_{i},t_{j}\right)A_{c}+E_{s}\sum_{j=1}^{i}\Delta \varepsilon_{c}\left(t_{i},t_{j}\right)A_{s}=0$$

Substituting Equation (5) into (10) yields:

(11) $$\left\{\sigma_{c}\left(t_{0}\right)\cdot n\cdot \varphi \left(t_{i},t_{0}\right)+n\cdot \sum_{j=1}^{i}\Delta \sigma_{c}\left(t_{i},t_{j}\right)\cdot [1+\chi \left(t_{i},t_{j}\right)\cdot \varphi \left(t_{i},t_{j}\right)]+E_{s}\cdot \varepsilon_{sh}\left(t_{i},t_{s}\right)-E_{s}\cdot \varepsilon_{sh}\left(t_{0},t_{s}\right)\right\}\cdot \rho \cdot A\;+\sum_{j=1}^{i}\Delta \sigma_{c}\left(t_{i},t_{j}\right)\cdot \left(1-\rho \right)\cdot A=0$$

Substituting Equation (3) into (11), the marginal stress increment of concrete is obtained:

When k = 1,

(12) $$\Delta \sigma_{c}\left(t_{i},t_{k}\right)=-\left\{N\left(t_{0}\right)\cdot n\cdot \rho \cdot \varphi \left(t_{i},t_{0}\right)+E_{s}\cdot \rho \cdot \left[1+\left(n-1\right)\cdot \rho \right]\cdot A\cdot \left[\varepsilon_{sh}\left(t_{i},t_{s}\right)-\varepsilon_{sh}\left(t_{0},t_{s}\right)\right]\right\}/\;\{\left(1-\rho \right)\cdot \left[1+\left(n-1\right)\cdot \rho \right]\cdot A+n\cdot \rho \cdot \left[1+\left(n-1\right)\cdot \rho \right]\cdot A\}$$

When k = 2,3,…,i

(13) $$\Delta \sigma_{c}\left(t_{i},t_{k}\right)=-\{N\left(t_{0}\right)\cdot n\cdot \rho \cdot \varphi \left(t_{i},t_{0}\right)+n\cdot \rho \cdot \left[1+\left(n-1\right)\cdot \rho \right]\cdot A\cdot \sum_{j=1}^{k-1}\Delta \sigma_{c}\left(t_{i},t_{j}\right)\cdot [1+\chi \left(t_{i},t_{j}\right)\cdot \varphi \left(t_{i},t_{j}\right)]\;+E_{s}\cdot \rho \cdot \left[1+\left(n-1\right)\cdot \rho \right]\cdot A\cdot \varepsilon_{sh}\left(t_{i},t_{s}\right)-E_{s}\cdot \rho \cdot \left[1+\left(n-1\right)\cdot \rho \right]\cdot A\cdot \varepsilon_{sh}\left(t_{0},t_{s}\right)+\left(1-\rho \right)\cdot \left[1+\left(n-1\right)\cdot \rho \right]\cdot A\cdot \sum_{j=1}^{k-1}\Delta \sigma_{c}\left(t_{i},t_{j}\right)\}/\;\{\left(1-\rho \right)\cdot \left[1+\left(n-1\right)\cdot \rho \right]\cdot A+n\cdot \rho \cdot \left[1+\left(n-1\right)\cdot \rho \right]\cdot A\}$$

where *k* is the number of tiny periods of time from the start of the load to the end of the load.

The iterative steps below are followed to calculate the long-term stress and strain of SRC columns using the iterative method:

1. Obtain $\Delta \sigma_{c}\left(t_{i},t_{1}\right)$ according to Equation (12);
2. Calculate an iteration according to Formula (13) to obtain $\Delta \sigma_{c}\left(t_{i},t_{2}\right)$,$\Delta \sigma_{c}\left(t_{i},t_{3}\right)$,…,$\Delta \sigma_{c}\left(t_{i},t_{i}\right)$ successively;
3. According to the results of step 1 and step 2, the cumulative increment of stress of concrete under long-term load $\sum_{j=1}^{i}\Delta \sigma_{c}\left(t_{i},t_{j}\right)$ can be obtained.
4. From Equation (8), the cumulative increment of stress of the steel bar under long-term loading can be described as follows:
  (14) $$\sum_{j=1}^{i}\Delta \sigma_{s}\left(t_{i},t_{j}\right)=-\frac{A_{c}}{A_{s}}\sum_{j=1}^{i}\Delta \sigma_{c}\left(t_{i},t_{j}\right)$$
5. According to Equation (10), the cumulative increment of strain produced by SRC in the process of shrinkage and creep is:
  (15) $$\sum_{j=1}^{i}\Delta \varepsilon_{c}\left(t_{i},t_{j}\right)=\sum_{j=1}^{i}\Delta \varepsilon_{s}\left(t_{i},t_{j}\right)=-\frac{\left(1-\rho \right)}{E_{s}\cdot \rho }\sum_{j=1}^{i}\Delta \sigma_{c}\left(t_{i},t_{j}\right)$$

## 3. Implementation of Programming

### 3.1. Prediction Model of Concrete Shrinkage and Creep

The shrinkage strain and creep coefficient of concrete are calculated using the modified prediction model proposed in [37].

The modified prediction model of concrete shrinkage strain is:

(16) $$\varepsilon_{sh}\left(t_{i},t_{s}\right)=\xi \left(t_{i}-t_{s}\right)\cdot \varepsilon_{cso}\cdot \beta \left(t_{i}-t_{s}\right)$$

where $\varepsilon_{cso}$ and $\beta \left(t_{i}-t_{s}\right)$ are the nominal shrinkage coefficient and the coefficient of shrinkage developing with time, respectively [38]; and $\xi \left(t_{i}-t_{s}\right)$ is the shrinkage correction factor of concrete. The time-varying law model of the shrinkage correction coefficient $\xi \left(t_{i}-t_{s}\right)$ of concrete is:

(17) $$\xi \left(t_{i}-t_{s}\right)=\{\begin{array}{ll} 0.326\cdot ln\left(t_{i}-t_{s}\right)+0.289 & ,\left(t_{i}-t_{s}\right)\leq 30d\\ 1.380 & ,\left(t_{i}-t_{s}\right)>30d \end{array}$$

The modified prediction model of the concrete creep coefficient is:

(18) $$\varphi \left(t_{i},t_{0}\right)=\eta \left(t_{i}-t_{0}\right)\cdot \phi_{o}\cdot \beta_{c}\left(t_{i}-t_{0}\right)$$

where $\phi_{o}$ and $\beta_{c}\left(t_{i}-t_{0}\right)$ are the nominal creep coefficient and the coefficient of creep developing with time after loading, respectively [38]; and $\eta \left(t_{i}-t_{0}\right)$ is the creep correction coefficient of concrete, taking 0.7387.

### 3.2. Programming Flowchart

**Figure 2:**
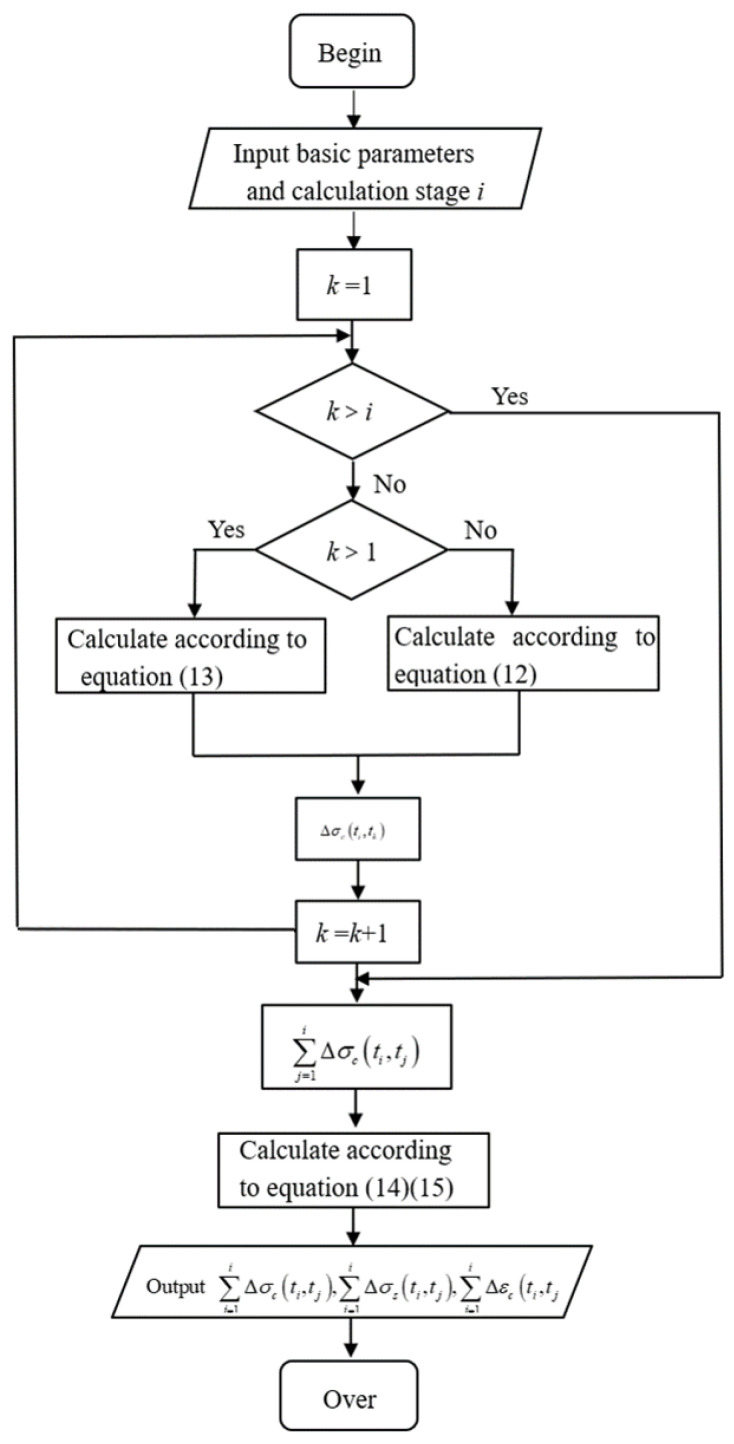
Program block diagram.

Based on the above iterative calculation steps, and combined with the modified prediction model of concrete shrinkage and creep [37], we used object-oriented Visual C++ 6.0 language as the programming language and apply a user-friendly visual interface to achieve the desired functionality. Thus, we created a long-term stress–strain calculation program for SRC axial compression columns that can be run on the Windows platform. The program block diagram is shown in Figure 2.

### 3.3. Example Verification

Ordinary SRC columns with bonded steel bars have a cross-section size of b×h = 0.25 m × 0.25 m, and four steel bars with a diameter of 14 mm are symmetrically arranged. The net section area of concrete $A_{c}$ is 61,885.0 ${\mathrm{mm}}^{2},$ and the area of steel bar $A_{s}$ is 615.0 ${\mathrm{mm}}^{2}$, the elastic modulus of concrete $E_{c}\left(t_{0}\right)$ and ordinary steel bar $E_{s}$ are 2.9 × 10^4^ MPa and 2.03 × 10^5^ MPa, respectively. The axial force is 520.0 kN while ignoring the dead weight of the column. The creep coefficient of concrete $\varphi_{\infty }$ is known to equal 3.0, and the shrinkage strain $\varepsilon_{sh\infty }$ is 200 με from the beginning of loading (t=0) to time t=∞. Calculate the stress distribution between the concrete and steel bars in the section from the beginning (t=0) loading to t=∞. The results of the calculations and the results of the classic example in [22] are shown in Table 1.

According to Table 1, the difference between the program calculation results of the concrete stress and the results in [22] is 0.02 MPa (a deviation of 0.33%), and the difference between the program calculation results of steel bar stress and the results in [22] is 8.11 MPa (a deviation of 3.54%). The maximum deviation of the results is only 3.54%, which shows that the program can accurately predict the long-term stress of SRC columns under axial compression, and the theoretical calculation formula derived in this paper produces high-accuracy results.

## 4. Experimental Validation

### 4.1. Experimental Setup

**Figure 3:**
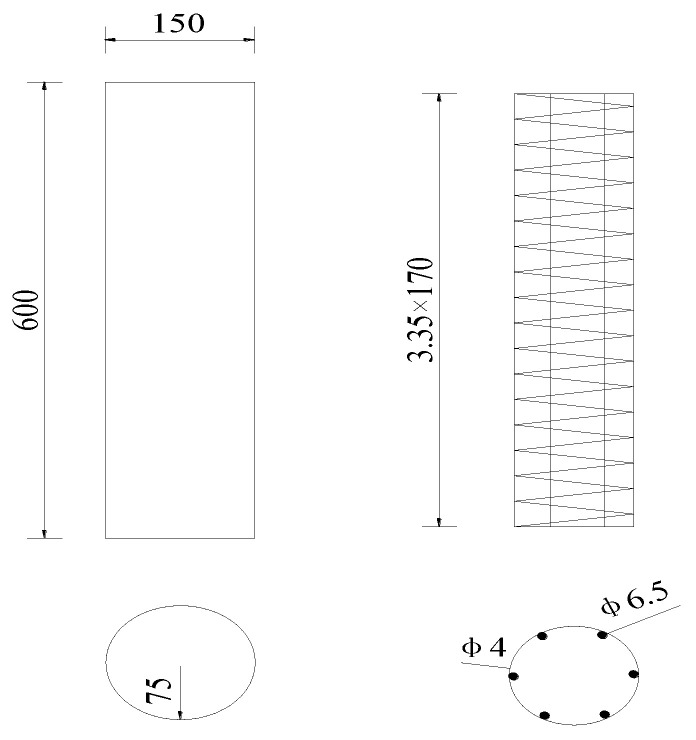
Size and steel bar of the shrinkage and creep columns (unit: mm).

The test adopts P.O 52.5 ordinary Portland cement, and the mix ratio of concrete is: cement:sand:gravel:water:water-reducing agent = 460:585:1175:232.5:3.68. Two plain concrete columns (numbered S1# and S2#,) and two SRC columns (numbered G1# and G2#) were made for the long-term deformation performance test of concrete columns. The height and diameter of the columns were 600 mm and 150 mm, respectively. The bar was made of the steel R235, and the reinforcement ratio was 1.13%. The size and steel bar of the shrinkage and creep column are shown in Figure 3. A jack and a pressure sensor were used to perform axial loading on the creep column on the creep frame, and the axial force was 159.1 kN, and the loading age $t_{0}$ was 21 days. During the long-term test, the axial force was kept constant.

**Figure 4:**
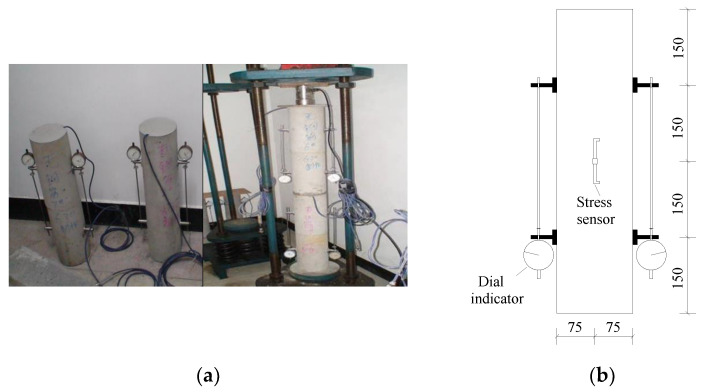
(**a**) Shrinkage and creep testing; (**b**) shrinkage and creep test apparatus (unit: mm).

Vibrating wire strain sensors were embedded in all concrete columns to test the shrinkage and creep deformation of the components, and mechanical dial indicators were installed symmetrically on the surface. The strain value of this experiment is mainly obtained by strain gauges. The dial indicators on both sides of the column are used to validate the values obtained by the strain gauges, and the value of the dial indicators is the average value. The test cycle was 750 days. The test is shown in Figure 4. The test was performed in a constant temperature and humidity environment with a temperature and relative humidity of 22.0 °C and 55.0%, respectively. The cubic compressive strength and elastic modulus of concrete at 28 days of age were 45.5 MPa and 30.8 GPa, respectively.

### 4.2. Comparison of Test Results and Program Calculation Results

**Figure 5:**
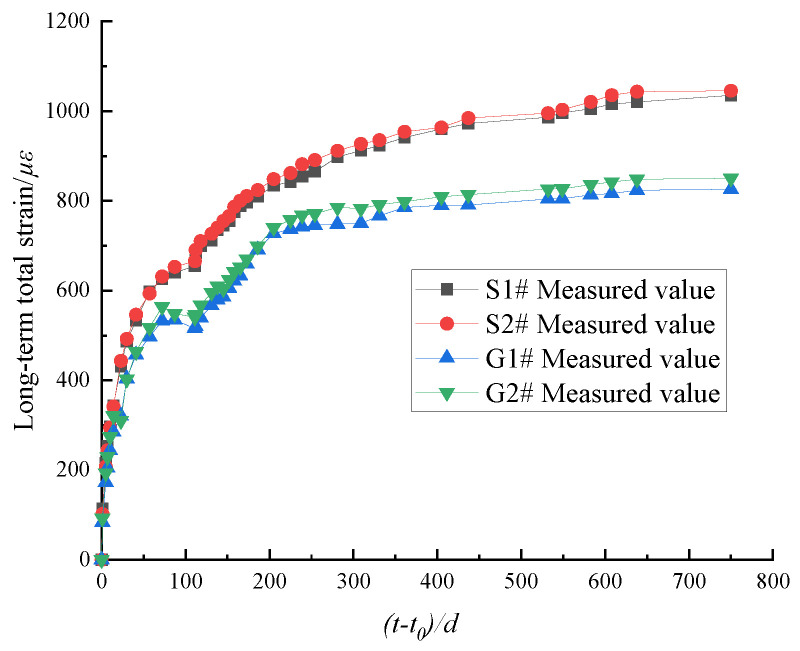
The long-term total strain of concrete columns.

Taking the loading age as the starting point, the long-term total strain test results of the concrete columns are shown in Figure 5.

Figure 5 shows that the test results of the plain concrete column and the SRC column exhibit similar trends with the load holding time $\left(t-t_{0}\right)$. The growth rate is high in the first 270 days, and strongly decreases after 270 days, the total strain gradually becomes stable. The strain of the plain concrete column is significantly larger than that of the SRC column. When the duration of loading $\left(t-t_{0}\right)$ is 750 days, the total strains of S1# and S2# are 1025.25 με and 1045.35 με, and the total strains of G1# and G2# are 825.69 με and 850.4 με. The maximum difference in the total strain between the plain concrete column and the SRC column is 26.60%. These results indicate that the steel bar had a strong influence on the long-term strain of the concrete column, and the steel bar constrained the shrinkage and creep of the concrete column, causing stress redistribution in the section.

**Figure 6:**
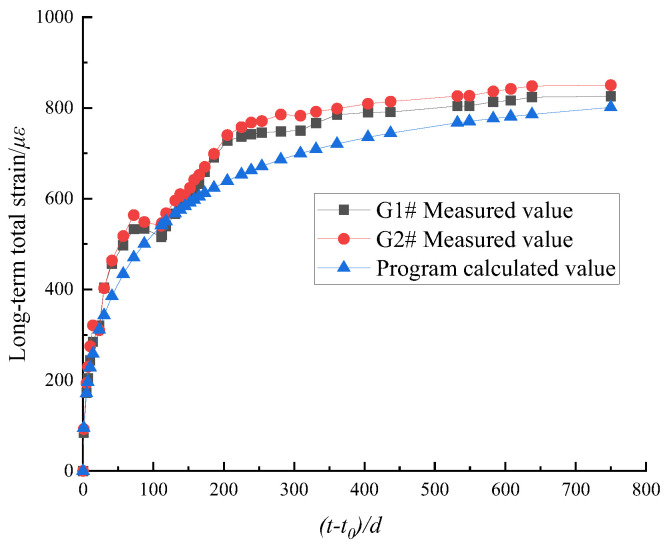
Comparison between calculated results and measured values.

The long-term strain analysis of SRC columns G1# and G2# was performed using the calculation program, and the calculated results were compared with the measured values. Taking loading age $t_{0}$ as the starting point, the comparison between calculated results and measured values of long-term total strain of SRC columns is shown in Figure 6.

Figure 6 shows that the results of the calculation program are basically consistent with the variation trend of the measured values with the load holding time $\left(t-t_{0}\right)$. The growth rate is high in the first 270 days, and strongly decreases after 270 days, the total strain gradually becomes stable. When the load holding time $\left(t-t_{0}\right)$ is 750 days, the calculated strain of the SRC column is 801.23 με, and the measured strains of the G1# and G2# columns are 825.69 με and 850.4 με, respectively. The errors between the calculated strain of the SRC column and the measured strain of the G1# and G2# columns are only 2.96% and 5.78%, respectively, which are small. These results show that the program based on the theory of long-term stress and strain calculation can accurately predict the long-term stress and strain of the SRC column.

## 5. Results and Discussion

### 5.1. Long-Term Stress

**Figure 7:**
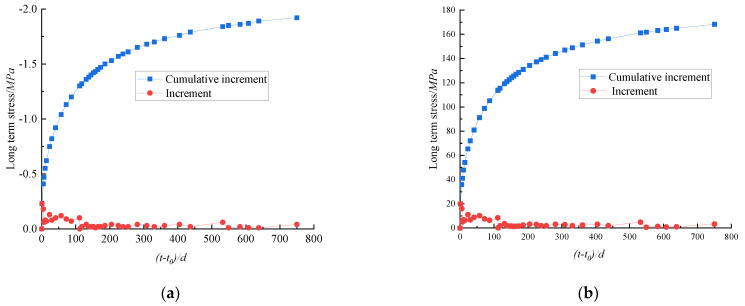
(**a**) Long-term increment change of concrete stress; (**b**) Long-term stress increment change of steel bar.

Taking the loading age as the starting point, the calculation program was used to analyze the long-term stress changes in Columns G1# and G2# within the observation period. The variation law of the stress or stress increment of the concrete and steel bars in each test period is shown in Figure 7.

These figures show that the long-term stress of concrete and steel bars exhibit similar trends with the load holding time $\left(t-t_{0}\right)$. The growth rate is high in the first 270 days, and strongly decreases after 270 days, the total stress gradually becomes stable. When the load holding time $\left(t-t_{0}\right)$ is 750 days, the concrete produces a long-term tensile stress increment of 1.92 MPa, the steel bar produces a long-term compressive stress increment of 168.26 MPa, and the total stress difference between the concrete and the steel bar reaches 98.86%, which is large. This result indicates that the steel bar had a strong influence on the long-term stress of the concrete column, and the steel bar constrained the stress of the concrete column, leading to stress redistribution in the section.

### 5.2. Long-Term Stress and Strain

**Figure 8:**
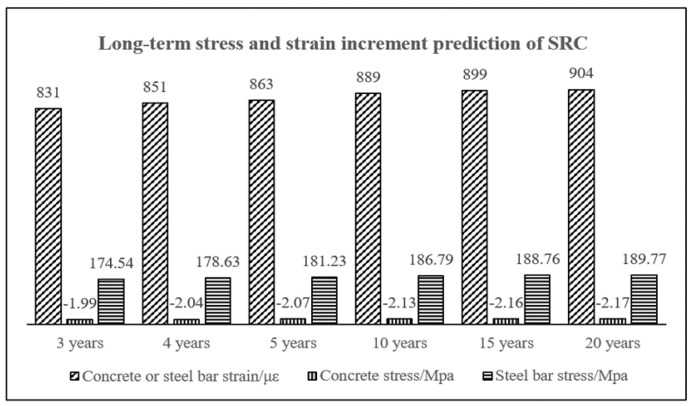
Long-term strain increment and stress increment prediction of columns # G1 and #G2.

To study the long-term stress and strain variation law of SRC columns, the calculation program was used to predict the long-term stress–strain increment of G1# and G2# columns. The prediction results of some test periods are shown in Table 2 and Figure 8.

Table 2 and Figure 8 show that after 3 years, the concrete stress decreases by 1.99 MPa, the steel bar stress increases by 174.54 MPa, and the strain of the concrete or steel bar increases by 831 με. Between 3 and 20 years, the stress and strain of the concrete and steel bars show a slowly increasing trend. The stress of concrete decreases by 0.18 MPa, the stress of the steel bar increases by 15.23 MPa, and the strain of the concrete or steel bar increases by 73 με. This result indicated that the stress and strain of the concrete and steel bars developed rapidly in the first three years, and then gradually slowed down.

## 6. Conclusions

Considering the effect of cross-section stress redistribution and each stress increment during long-term stress and strain of SRC columns, the stress and strain calculation formula of SRC axial compression columns under long-term load was derived. Using the object-oriented Visual C++ language, the stress and strain calculation program of the axial compression column of SRC under long-term load was programmed. The following conclusions can be drawn based on the results of this study:

- The theoretical calculations agreed with the results of classic examples by the study verification. Therefore, the derived formula for the stress and strain of the SRC axial compression column under long-term loading and the calculation program were more accurate.
- An experimental study of the long-term deformation performance of concrete columns under a constant temperature and humidity environment was performed to validate the proposed theoretical formulae. The long-term strain of plain concrete and SRC shows the same trend with load holding time. The growth rate was high in the first 270 days and strongly decreases after 270 days, the total strain gradually becomes stable. The strain of the plain concrete column was significantly larger than that of the SRC column. When the load holding time was 750 days, the maximum difference in total strain between the plain concrete column and the SRC column reached 26.60%, which was large. Therefore, the steel bar constrains the shrinkage and creep of the concrete column.
- The calculated results of the program are consistent with the trends of the measured strains with the load holding time. The strain growth rate was high in the first 270 days and strongly decreases after 270 days, the total strain gradually becomes stable. When the load holding time was 750 days, the errors between the calculated strains of the program and the measured strains of the G1# and G2# columns were only 2.96% and 5.78%, respectively. The program based on the theory of long-term stress and strain calculation can thus accurately predict the long-term stress and strain of the SRC axial compression column.
- The long-term stresses of columns G1# and G2# were analyzed by the calculation program. The long-term stresses of the concrete and steel bars show the same trends as the load-holding time. The stress growth rate was high in the first 270 days and strongly decreases after 270 days, the total stress gradually becomes stable. When the load holding time was 750 days, the difference in stress between the concrete and steel bar was large, which indicates that the steel bar has a strong influence on the long-term stress of the concrete column, and the steel bar constrains the stress of the concrete column, causing stress redistribution in the section. The long-term strain and stress increment of columns G1# and G2# were predicted by the calculation program. In the first 3 years, the stress and strain of the concrete and steel bars developed rapidly and then gradually slowed down.

## Figures and Tables

**Table 1:** Stress changes in SRC columns.

Calculated Age	t=0		t=∞	
	Concrete Stress/MPa	Steel Bar Stress/MPa	Concrete Stress/MPa	Steel Bar Stress/MPa
Reference [22]	7.86	55.02	6.13	229.10
Procedure	7.86	55.02	6.11	237.21

**Table 2:** Long-term strain increment and stress increment prediction of columns #G1 and #G2.

$\mathrm{Duration}\;\mathrm{of}\;\mathrm{Drying}\;(t-t_{s})$	3 Years	4 Years	5 Years	10 Years	15 Years	20 Years
concrete or steel bar Strain/με	831	851	863	889	899	904
concrete stress/MPa	−1.99	−2.04	−2.07	−2.13	−2.16	−2.17
Steel bar stress/MPa	174.54	178.63	181.23	186.79	188.76	189.77

## Data Availability

Data are contained within the article.
